# Associations between SNPs in candidate immune-relevant genes and rubella antibody levels: a multigenic assessment

**DOI:** 10.1186/1471-2172-11-48

**Published:** 2010-10-05

**Authors:** V Shane Pankratz, Robert A Vierkant, Megan M O'Byrne, Inna G Ovsyannikova, Gregory A Poland

**Affiliations:** 1 Mayo Clinic Division of Biomedical Statistics and Informatics, Harwick 7 200 1st Street SW Rochester, MN 55905 USA; 2 Mayo Clinic Division of Biomedical Statistics and Informatics, Charlton 6 200 1st Street SW Rochester, MN 55905 USA; 3 Mayo Clinic Vaccine Research Group Program in Translational Immunovirology and Biodefense 200 1st Street SW Rochester, MN 55905 USA

## Abstract

**Background:**

The mechanisms of immune response are structured within a highly complex regulatory system. Genetic associations with variation in the immune response to rubella vaccine have typically been assessed one locus at a time. We simultaneously assessed the associations between 726 SNPs tagging 84 candidate immune response genes and rubella-specific antibody levels. Blood samples were obtained from 714 school-aged children who had received two doses of MMR vaccine. Associations between rubella-specific antibody levels and 726 candidate tagSNPs were assessed both one SNP at a time and in a variety of multigenic analyses.

**Results:**

Single-SNP assessments identified 4 SNPs that appeared to be univariately associated with rubella antibody levels: rs2844482 (p = 0.0002) and rs2857708 (p = 0.001) in the 5'UTR of the LTA gene, rs7801617 in the 5'UTR of the IL6 gene (p = 0.0005), and rs4787947 in the 5'UTR of the IL4R gene (p = 0.002). While there was not significant evidence in favor of epistatic genetic associations among the candidate SNPs, multigenic analyses identified 29 SNPs significantly associated with rubella antibody levels when selected as a group (p = 0.017). This collection of SNPs included not only those that were significant univariately, but others that would not have been identified if only considered in isolation from the other SNPs.

**Conclusions:**

For the first time, multigenic assessment of associations between candidate SNPs and rubella antibody levels identified a broad number of genetic associations that would not have been deemed important univariately. It is important to consider approaches like those applied here in order to better understand the full genetic complexity of response to vaccination.

## Background

The importance of developing protective humoral immunity following vaccination is widely recognized, as those who fail to respond are at increased risk of contracting the disease if exposed. Rubella is well controlled via vaccination programs in industrialized countries, but epidemics of the disease occasionally occur in developing countries and both rubella virus infection and congenital rubella syndrome remain a major health concern around the world [1,2]. Understanding how host genetic influences modify response to rubella immunization may shed light into the biology of immunity to rubella infection, as well as into the potential development of even more highly effective vaccines. While the heritability of antibody responses to rubella vaccination has been estimated to be as high as 46% [3], knowledge of the genetic control of rubella vaccine-induced immunity remains incomplete.

Our group and others have shown that polymorphisms in the human leukocyte antigen (HLA) region, as well as SNPs in cytokine and cytokine receptor genes, are associated with differences in a variety of immune responses to rubella vaccine, but do not explain all of the variance in immune responses seen within the population [4-16]. Studies with other viral vaccines, such as measles and mumps, have demonstrated associations between cytokine and cytokine receptor gene polymorphisms and immune responses [17,18]. Because of the central role of cytokines as intercellular protein messengers and the role of their receptors in the immune response cascade, cytokine and cytokine receptor gene polymorphisms may significantly influence the outcome of rubella vaccine immune response. For example, polymorphisms in both coding and noncoding regions of these genes can affect multiple aspects of cytokine biology, such as transcriptional activity protein production, receptor binding and functional activity [19,20]. Thus, a wide variety of genes is likely to be important in regulating immune response to live viral vaccines.

While the role of cytokines in antiviral immune responses has been established, little is known about how other gene families control immune responses to rubella virus. Studies in a variety of other models (viruses, bacteria, microbial antigens) have also recently demonstrated the importance of innate and vitamin receptor genes in regulating immune responses [21-23]. In this regard, innate antiviral factor TRIM, toll-like receptors (TLR) and their associated intracellular signaling molecules activation is critical to stimulating innate and adaptive immunity [24]. Importantly, innate pathways detect infection and serve two purposes: mediate initial anti-viral response and prime more powerful and specific adaptive responses. Finally, vitamins and their receptors are known to have hormone-like attributes and were also found to affect innate and adaptive immunity [11,25]. To further characterize the impact of immune gene polymorphisms on variability in vaccine-induced humoral immunity, it is crucial to broadly examine variants in key genes important to the immune response to viral vaccines such as rubella. The genetic diversity of innate, adaptive, antiviral effector and other immune response genes has not been comprehensively studied within the context of rubella vaccine-induced humoral immune responses.

The discovery of genetic variations caused by single nucleotide polymorphisms (SNPs) has led to population-based immunogenetic studies intended to elucidate the potential relationship between host genomic variation and immune response [26]. A high level of regulatory complexity is required in the human immune system to insure a high probability of functional redundancy in both cell-mediated and humoral immune responses to vaccination [27,28]. For instance, one gene may be able to compensate for potential loss of function due to genetic variation in another. This would lead to reduced power to detect real associations because variation in immune response may only be apparent within subjects with genetic variants in both of the genes. Because such associations could be missed with single SNP analyses, when associations between genetic variants and measures of immune response are studied it is important to examine more than just their relationships with individual SNPs. That is, not only should genetic associations be studied for the main effects of single genetic variants on immune response, but multigenic associations, whether epistatic or otherwise, should be examined as well [29,30]. A growing collection of approaches is becoming available for the study of multigenic associations in association with disease states. These include relatively standard statistical approaches to study interactions, but also newer approaches that have been designed to search for the presence of multigenic effects in high dimensional genetic data consisting of many SNPs [31-38].

In this study, we searched for evidence of multigenic associations among a broad collection of SNPs identified from non-HLA genes that are candidates for being immunologically relevant to the development of a humoral response to rubella vaccination after two doses of measles-mumps-rubella vaccine. Due to the large number of genes in immune response gene families, we selected a group of key genes for each family, which, based on biologic plausibility and the published literature, are highly likely to be involved in regulating rubella immunity. We were particularly interested in assessing the degree to which the inclusion of multilocus associations would identify important genetic variation that contributes to rubella-specific antibody levels. To date, no such data have been published to address this issue.

## Results

We attempted to genotype a total of 768 SNPs in a cohort of 738 children who had received two doses of rubella vaccine. After SNPs with call rates below 90%, HWE p-values less than 0.001 or minor allele frequencies less than 5% were excluded from analysis, a total of 726 remained, for an overall genotyping success rate of 94.5%. Subject exclusions were made on the basis of poor DNA quality (n = 6), complete genotyping failure (n = 4) and call rates below 95% (n = 14). These exclusions removed 24 subjects, leaving 714 subjects for analysis. Data from these individuals are summarized in Table 1. Included with these summaries are the associations between the various descriptive variables and rubella antibody levels. Of note, differences in antibody levels were observed between males and females (p = 0.009), and among categories defined by the age at which the second rubella vaccination was received (p = 0.001).

**Table 1:** Characteristics of the study population and their associations with rubella-specific IgG antibody levels (reported in IU/ml).

Variable	Level	Number of subjects	Median	Q1 - Q3^a^	P-value^b^
Overall		714	34.5	19.2 - 63.7	
Age at enrollment (years)	11-13	212	37.0	19.1 - 67.1	0.967
	14-15	190	34.8	19.4 - 58.5	
	16-17	200	33.3	19.2 - 70.2	
	18-19	112	34.5	19.2 - 57.9	
Age at first rubella vaccination (months)	≤14	89	35.4	19.6 - 59.9	0.556
	15	384	31.4	19.0 - 62.2	
	16-17	119	41.2	18.9 - 72.6	
	≥18	122	39.9	21.8 - 66.2	
Age at second rubella vaccination (years)	≤5	205	27.6	16.6 - 55.3	0.001
	6-10	109	37.0	20.5 - 69.6	
	11	122	34.9	18.9 - 65.4	
	≥12	278	39.9	22.1 - 69.7	
Gender	Female	336	39.9	20.0 - 69.7	0.009
	Male	378	30.9	18.3 - 56.7	
Race	Other	65	33.4	17.7 - 62.9	0.903
	White	649	34.5	19.2 - 63.7	

**Notes**:

^a ^Q1 and Q3 represent the first (25^th ^percentile) and third (75^th ^percentile) quartiles, respectively.

^b ^p-value represents a test of the null hypothesis that there are no differences in the level of rubella antibody IgG levels among the categories represented.

**Figure 1:**
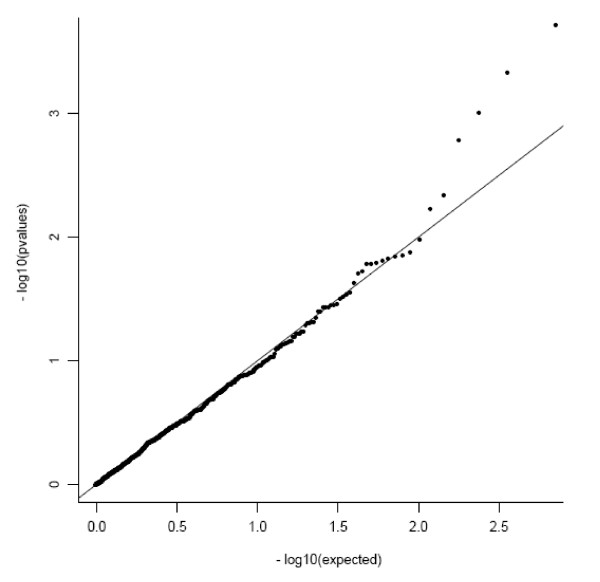
**Quantile plot of observed versus expected p-values (on the logarithmic scale) for single-SNP associations with rubella antibody levels**. The points in the upper right hand side of the plot that are distant from the line of unity correspond to the SNPs with the strongest evidence for association.

Figure 1 contains a quantile plot of the single-SNP p-values, each assessed individually. This figure suggests that there are a number of SNPs associated with rubella antibody levels. In particular, the top four SNPs stand out as being the most likely to be univariately associated with antibody levels, although their p-values were not smaller than the cut-off corresponding to a Bonferroni correction for 726 statistical tests. These SNPs were rs2844482 (p = 0.0002) and rs2857708 (p = 0.001) in the 5'UTR of the LTA gene, rs7801617 in the 5'UTR of the IL6 gene (p = 0.0005), and rs4787947 in the 5'UTR of the IL4R gene (p = 0.002). The minor allele of each of these SNPs was associated with increasing levels of rubella-specific antibody.

Our omnibus test of association between the number of positively associated alleles carried across all of these candidate SNPs and the observed antibody levels suggested that, even with the high number of null SNPs apparent from the univariate analyses, there was a significant association between this collection of SNPs and rubella antibody after performing a randomization test to account for multiple comparisons (p = 0.029). In order to narrow the list of SNPs that contributed to this omnibus association, we performed a stepwise selection procedure. This procedure identified a list of 29 SNPs that all contributed to the genetic association with rubella antibody levels when considered simultaneously in the same regression model, with a global p-value obtained via a randomization test of 0.017. These SNPs are illustrated in Table 2. The SNP with the smallest p-value in the univariate analyses, rs2844482, also had the smallest p-value after adjusting for the other SNPs in the multi-allelic model. Interestingly, several SNPs without a clear ordinal trend in univariate analyses were significantly associated with the outcome on a per-allele basis after controlling for other SNPs in candidate multi-allelic model. One example of this is rs1800795, which displayed a u-shaped trend across the three genotypes when examined individually (p = 0.511), and displayed a negative dose-response after controlling for other SNPs in the multi-allelic model (Allelic Fold-change, 95% CI: 0.79, 0.71-0.88, p < 0.001).

**Table 2:** SNP associations identified through the model selection procedure, sorted from the smallest to the largest multi-SNP p-value.

SNP	Gene	Location	MAF (%)	Median (Q1 - Q3)^a^			Univariate p-value	Multi-SNP Results		
				**0 Minor Alleles**	**1 Minor Allele**	**2 Minor Alleles**		**Estimate**^**b**^	**S.E.**^**c**^	**p-value**
rs2844482	*LTA*	5'UTR	15.5	32(18.6-59.3)	41.2(21.3-72.6)	72.5(41.5-108.7)	0.000	0.164	0.027	<0.001
rs1880241	*IL6*	5'UTR	49.1	30.1(17.6-59)	36.2(19.8-67.6)	40.3(20.3-68.2)	0.014	0.133	0.024	<0.001
rs2243248	*IL4*	5'UTR	7.3	36.2(19.4-66.2)	27.8(16.5-56.2)	17.9(17.8-129.9)	0.023	-0.182	0.036	<0.001
rs1800795	*IL6*	5'UTR	42.8	37.2(19.2-70.1)	32.6(19-61.4)	39.9(19.4-63.1)	0.511	-0.102	0.025	<0.001
rs2256965	*LST1*	intron	42.9	36.3(18.8-66.7)	33.9(18.6-64.5)	32.6(21.2-59.4)	0.891	0.085	0.021	<0.001
rs4787947	*IL4R*	5'UTR	8.7	32.7(18.4-62.4)	43.2(26.4-68.8)	79.4(48.5-107)	0.002	0.119	0.029	<0.001
rs2227284	*IL4*	intron	31.1	31.8(18.9-60.1)	37.9(19.6-69.5)	38.1(18.3-69.7)	0.179	0.081	0.020	<0.001
rs1800629	*TNF*	5'UTR	17.4	33.7(19.2-61.7)	39.5(19.6-73.5)	31.2(21.8-68.2)	0.132	0.104	0.026	<0.001
rs9610	*IL10RA*	3'UTR	44.1	39.9(21.3-69.7)	33.2(18.6-66.3)	29.6(18.3-52.4)	0.004	-0.064	0.017	<0.001
rs2256774	*IL2RA*	intron	33.8	33.6(18.1-59.2)	33.1(19.5-64.5)	42.8(26.1-78.7)	0.016	0.064	0.017	<0.001
rs9427092	*ADAR*	3'UTR	22.7	33.3(19-61.1)	34.8(19.5-67.5)	40.3(22.8-83.2)	0.102	0.069	0.021	0.001
rs2243300	*IL4*	5'UTR	8.3	33.8(19-63.3)	37.7(20.1-63.7)	46.7(29.1-262.1)	0.252	0.100	0.032	0.002
rs1153592	*RARB*	intron	16.4	36.6(19.6-65.4)	31.1(18.1-60.1)	41.4(20.5-61.1)	0.079	-0.067	0.023	0.003
rs228979	*IL2RB*	intron	26.0	37.3(20.6-66.5)	31.2(18-59.7)	25.9(18.8-67.6)	0.048	-0.053	0.019	0.005
rs3091338	*IL3*	3'UTR	38.2	33.3(18.2-60.6)	33.6(19.5-63.1)	39.8(20.7-72.3)	0.100	0.049	0.018	0.006
rs12757998	*RNASEL*	3'UTR	28.0	33.4(18.8-58.9)	37(19.6-68.3)	35.5(18.7-72.6)	0.130	0.052	0.019	0.006
rs1732778	*OAS2*	3'UTR	25.1	32.9(19.2-57.4)	38.4(19.1-71.7)	37.8(19.3-74.8)	0.036	0.052	0.019	0.007
rs4648212	*EIF2AK2*	intron	6.5	36.7(19.5-65)	27.6(16.6-52)	22.7(20.5-23.6)	0.018	-0.087	0.033	0.008
rs2229857	*ADAR*	coding	27.4	31.2(18-63)	40.3(20.5-65)	39.1(21.5-64.5)	0.072	0.049	0.019	0.011
rs17882988	*TNFRSF1B*	intron	17.3	34(18.8-62)	33.3(19.7-64.5)	67.8(33.2-108.1)	0.183	0.055	0.022	0.011
rs3740996	*TRIM5*	coding	10.7	37.3(19.6-68.2)	27.3(17.6-56.2)	39.8(21.1-81.9)	0.016	-0.069	0.027	0.011
rs2246614	*MUPCDH*	coding	34.9	36.3(20-68.2)	32.9(18.9-60.1)	35.3(17.7-64.6)	0.202	-0.043	0.018	0.014
rs2179	*TRIM22*	intron	30.0	37.4(19.6-66.3)	33.5(18.2-64.8)	26.8(17.2-46.7)	0.039	-0.044	0.018	0.015
rs2287350	*EIF2AK2*	intron	38.1	33.3(19.5-68.3)	36.1(19-67.2)	31.1(18.8-53.1)	0.057	-0.041	0.017	0.018
rs1422876	*IL12B*	5'UTR	48.9	31(17.9-66.2)	36.9(19.6-63.7)	36.3(19.5-62.2)	0.200	0.040	0.017	0.018
rs2226299	*IFNAR1*	intron	18.6	33.2(19.2-62.9)	35(18.2-67.6)	39.1(28.2-60.1)	0.308	0.049	0.021	0.022
rs6793694	*RARB*	intron	37.7	37.4(19.9-66.5)	33.3(18.8-63.1)	32.7(17.3-56.8)	0.039	-0.040	0.018	0.023
rs11064145	*SCNN1A*	3'UTR	42.5	28.4(17.9-61.1)	39.7(20.6-67.6)	36.5(19.1-60.1)	0.100	0.038	0.017	0.024
rs12626735	*TMEM50B*	3'UTR	21.1	38.4(19.6-69.7)	29.6(18.3-55.8)	31.2(18.9-59.1)	0.013	-0.045	0.020	0.030

Identified SNPs are all significant when adjusting for all other SNPs shown in the table, and for the descriptive variables outlined in Table 1. Univariate results are shown for comparison.

**Notes**:

^a ^Q1 and Q3 represent the first and third quartiles, the 25^th ^and 75^th ^percentiles, respectively.

^b ^Estimate represents the difference in log(antibody level) associated with carrying one copy of the minor allele. The anti-log of this quantity provides an estimate of the fold-change in antibody levels associated with carrying one copy of the minor allele.

^c ^S.E. represents the standard error of the Estimate. The ratio of the Estimate to the S.E. provides a measure of the relative strength of the effect among the SNPs

**Figure 2:**
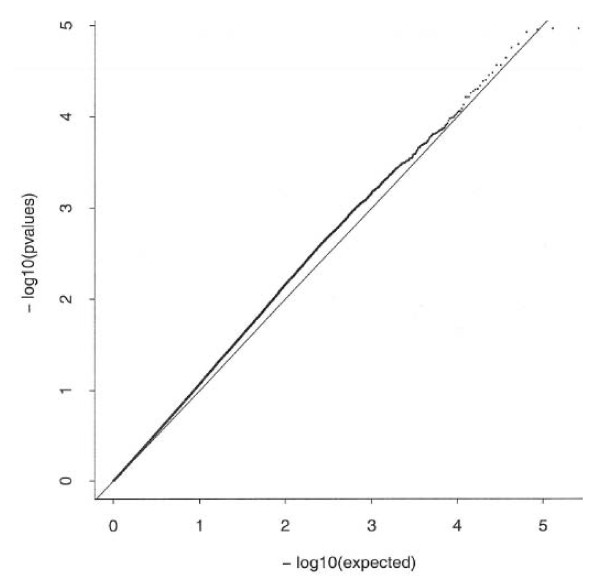
**Quantile plot of observed versus expected p-values (on the logarithmic scale) for the associations between the combinations formed from each possible pair of SNPs and rubella antibody levels**. While the observed p-values depart somewhat from the line of unity, they do not follow a pattern that suggests the presence of major epistatic effects.

The plot in Figure 2 represents the results from the analysis of the associations between the 263,175 possible pair-wise combinations of SNPs and rubella antibody levels. The observed p-values deviate somewhat from the line of unity, but not in a way that is indicative of the presence of a small number of pairs of SNPs that are strongly associated with rubella antibody levels. In fact, the smallest p-value of 1.07 × 10^-5 ^for the combination of rs10489626, an intronic SNP in IL12RB2, and rs1420094, a 3' flanking SNP in IL18R1, was actually less extreme than what would be expected if there were no pair-wise epistatic associations present.

As with the assessment of all possible pair-wise interactions, the use of recursive partitioning algorithms did not yield strong evidence in favor of major epistatic interactions. After forming the recursive partitioning tree, an assessment of the degree of pruning required to avoid over-fitting indicated that the best-fitting tree was one with a single node, with the split being on rs7801617. Because of this finding, further analyses seeking additional multi-allelic epistatic effects were not pursued.

## Discussion

Because of the regulatory complexity of immune responses to complex antigens, there is almost certainly a diversity of genes that together influence the immune response to vaccination. While there are many genes that are strong candidates, the bulk of those that have an effect on the levels of rubella-specific antibody raised in response to immunization have yet to be identified. In this study, we examined a broad collection of SNPs that tag 84 genes that have been identified as potentially playing a role in the immune process. We have performed an array of analyses in an attempt to identify potential multigenic associations between these candidate SNPs and the observed levels of rubella-specific antibody levels following MMR-II vaccination.

As we have reported previously, a number of SNPs and HLA alleles have already been identified as being univariately associated with variation in responses to rubella immunization [7-16]. In the analyses presented here, we were able to examine all univariate SNP associations with rubella antibodies simultaneously. This assessment suggested that the top four SNPs were among those that were significantly associated with the humoral immune response. When broader analyses were performed that explicitly searched for multiple SNPs that independently contributed to variation in rubella antibody levels while statistically correcting for multiple testing using a randomization approach, we found that there was a significant association between the number of positively associated alleles carried by an individual across all candidate SNPs and differences in rubella antibody level (p = 0.029). In order to identify the subset of SNPs that captured this signal, we subsequently performed a SNP selection procedure and identified a total of 29 SNPs that simultaneously provided information on the levels of rubella-specific antibodies (p = 0.017). In both of these analyses, we employed resampling procedures to account for the process of model building and selection as the tests for significance were performed [39].

We performed two separate analyses in our search for epistatic effects among the 726 SNPs. We first assessed the significance of all possible two-way combinations of SNPs (263,175 statistical tests). While there were more SNP pairs with p-values between 0.01 and 0.0001 than would have been expected under the null hypothesis, we found that the smallest p-value was actually larger than what would be expected under the hypothesis that no SNP pairs were significantly associated with rubella antibody levels. Likewise, as we performed a recursive partitioning analysis, we found that the best-fitting classification tree had only one node. This provided further evidence against the presence of major epistatic associations being in effect among the SNPs from the candidate genes under study.

While the evidence for epistasis among the candidate SNPs is not strong, our analysis supports the concept that considering the broader collection of a large number of SNPs does provide additional insight into the genetic control of rubella antibody levels. By assessing all SNPs in a single model we were able to identify a broader collection of candidates than was possible when each SNP was considered individually. While the univariate tests implicated SNPs in the LTA, IL6 and IL4R genes, the analysis of all SNPs identified not only these genes but also a broader collection of genes that appear to contribute to variation in rubella antibody levels. The 29 SNPs that were jointly associated with humoral immunity are shown in Table 2. These SNPs that were jointly associated with differences in rubella antibody levels resided in a total of 23 genes.

While there is evidence in favor of there being a major genetic component to the observed levels of rubella-specific antibodies following vaccination [3], the current knowledge of the genetic control of immune responses to rubella immunization has been obtained from studies that focused on a small number of genetic loci that were analyzed one at a time [7-9,11-14]. Given the genetic diversity required by the immune system to mount an adequate response to a wide number of pathogens, it is likely that these one-at-a-time approaches are inadequate for the comprehensive study of the genetic correlates of immune response. Indeed, the efforts reported here implicated a broader number of candidate genes as potentially being involved in modulating rubella-specific antibody levels using multigenic analyses that examined more than one locus at a time.

The genes that have been implicated as being associated with rubella antibody levels were selected for study because of their general relevance to viral immunity. The humoral immune response to rubella vaccine reflected in assayed antibody levels is the cumulative result of the actions and interactions of multiple genes and pathways. Those SNPs that were identified as being important for humoral immunity to rubella are included in Table 2. Among these genes are cytokine (Th1/Th2/inflammatory) and cytokine receptor, lymphotoxin alpha (LTA), leukocyte specific transcript-1 (LST1), antiviral effector (OAS2), IFN-type I-induced (EIF2AK2), adenosine deaminase (ADAR), vitamin A receptor family (RARB), the innate antiviral factor (TRIM5 and TRIM22), mucin-like protocadherin (MUPCDH) and other genes. These identified variants reside in genes spanning a broad collection of different classes of immune-related genes. Since genetic polymorphisms may function jointly to determine the outcome of vaccine-induced antibody response, it is reasonable to suggest that the observed antibody level effects in our study may be an outcome of combinations of SNP-defined alleles and immune response pathways, with the cumulative sum of variants across these genes influencing levels of rubella-specific antibodies. Better understanding the immunogenetic impact of multiple gene family pathways critical to development of humoral immune responses following rubella vaccination may provide insight into the factors that influence rubella immunity.

### Strengths and Limitations

This manuscript outlines the first analysis of its kind, an assessment of the associations between a large collection of SNPs from immunologically relevant genes and rubella antibody levels. This analysis made it possible to examine all SNPs we genotyped on a well-characterized cohort of study participants and assess the likelihood that the univariate SNP results were significant on their own. This examination also made it possible to study a variety of types of associations between candidate SNPs and rubella antibody levels, both epistatic and otherwise. When complicated analytical approaches were employed, we used resampling methodologies to obtain statistical significance levels that were not over-trained to the data [39]. This study does have two important limitations, however. First, as with all genetic association studies there is a need for replication, and a replication cohort is currently unavailable to us. Second, there was limited power to detect significant epistatic associations; for two SNPs with MAFs of 0.15 there was 80% power to detect interactions if the effect of carrying one copy of each of the minor alleles was associated with a 1.8-fold departure from additivity, while the detectable effect for a main effect for these SNPs was for a 1.25-fold difference. An additional limitation of this study is that our analyses were restricted to a collection of 726 tagSNPs identified from 84 candidate genes. There are likely to be other genetic variants that contribute to differences in rubella antibody levels that we have not assessed.

## Conclusion

The implications of this work are two-fold. First, it presents a novel methodological approach that can provide additional insights into candidate genetic associations when analyzing similar data in other studies. When searching for associations between genes that are part of a complex system and outcomes that are potentially influenced by genetic variation in this system, it is important to consider more than just simple single-SNP assessments. Second, it is clear that the "dominant allele" model of genetic regulation of complex system processes such as immune response is both uncommon and too limiting. At least for those viral vaccines we have studied (measles, mumps, rubella, vaccinia and influenza), variation in immune responses are clearly the result of small contributions from many genes and sets of genes acting in concert. These data provide motivation to move beyond simple univariate associations to the types of analyses illustrated in this report in order to identify significant multigenic associations otherwise unobservable by simple univariate models. The value of approaches such as this include the ability to more clearly identify and model the genetic determinants of immune response to viral vaccines. The findings from these models can then be used to inform the development of next-generation vaccines.

## Methods

### Study subjects

The characteristics of the 738 healthy children and young adults (age 11 to 19 years) who participated in this study have previously been described [40]. Study subjects were immunized with two age-appropriate doses of live measles-mumps-rubella-II (MMR-II) vaccine containing the Wistar RA 27/3-strain of rubella virus. The study participants were residents of Olmsted County, Minnesota, a community where no case of rubella infection had been reported during their lifetimes. The majority of the study population was white (91%), with 47% being female, with a median age at enrollment of 15 years (Table 1). The median age at the first and second immunization were 15 months and 11 years, respectively, and the median time between last rubella immunization and sample draw was 5.8 years. While 738 children and young adults were enrolled in the study, after exclusion for genotyping quality a total of 714 subjects were retained for analyses in this study. The Mayo Clinic Institutional Review Board granted approval for the study, and written informed consent (parental permission and assent for minors) was obtained.

### Humoral Immunity Assays

Rubella-specific IgG antibodies after two doses of the rubella vaccine were detected in serum by a whole rubella virus-specific chemiluminescent immunoassay (Beckman Coulter Access, Fullerton, CA) according to the manufacturer's instructions. The limit of detection for this assay was 0.5 IU/ml and the coefficient of variation in our laboratory was 6%.

### TagSNP selection

We selected a total of 768 tagSNPs from 84 candidate genes encoding cytokines and cytokine receptors. The details of SNP selection have described previously [12]. Briefly, we generated a list of SNPs within, and 10 kb upstream and downstream of, the targeted candidate genes using the Hapmap Phase II (http://www.hapmap.org)[41], Seattle SNPs (http://pga.mbt.washington.edu/)[42], and NIEHS SNPs (http://egp.gs.washington.edu/)[43] as source databases. We included SNPs that had validation data, successful predictive genotyping scores for Illumina GoldenGate assays, and reported minor allele frequencies (MAF) ≥0.05 in the list of possible SNPs. We used the linkage disequilibrium (LD) based ldSelect algorithm [44] to identify tagSNPs from the initial list with pairwise LD threshold of r^2 ^<0.90 for Caucasians. Using these criteria, we selected 768 potential SNPs in our candidate genes of interest (see Additional file 1). We used the nomenclature described by den Dunnen and Antonarakis for all genotype variants [45].

### Genotyping methods

Our genotyping methods were previously described in detail [17]. Briefly, genomic DNA samples (n = 738, 250 ng each) obtained from frozen blood clots using the Puregene extraction kit (Gentra Systems Inc., Minneapolis, MN) were genotyped for 768 candidate SNPs using a custom designed 768-plex Illumina GoldenGate™ assay (Illumina Inc., San Diego, CA), following the manufacturer's instructions [12]. All the SNPs selected for the custom Illumina panel had design scores >0.4. A Coriel Trio DNA (mother: NA11875, father: NA10859, daughter: NA10858) and two other genomic DNA controls were used as standards to review and refine clustering. These controls were genotyped on each plate, which allowed us to assess genotyping concordance of replicate subjects.

We used PCR-based TaqMan assays (Applied Biosystems, Foster City, CA) as the secondary platform to genotype SNPs that failed genotyping on the Illumina platform. All assays were performed according to the manufacturer's instructions, and the results were analyzed on the ABI Prism 7900 using Sequence Detection Software (Applied Biosystems).

Illumina 10% GenCall scores >0.4 and call rates >90% were used as thresholds for the initial laboratory quality control. The data from genotype calls made by BeadStudio 2 software were transferred to SAS for further analysis, where call rates for each subject, and for each SNP, were estimated. Participants' genotypes were used to estimate allele frequencies for each SNP, and departures from Hardy-Weinberg equilibrium (HWE) were assessed using a Pearson goodness-of-fit test or, for SNPs with a minor allele frequency of less than 5%, a Fisher exact test [46].

### Statistical methods

The purpose of the efforts reported here was to determine the multigenic contributions of SNPs from the candidate genes involved with immunity that were selected for this study. Prior to performing statistical comparisons, the characteristics of study participants were categorized and examined in relationship to the measured rubella-specific IgG antibody levels (represented as IU/ml). The median, and the 25^th ^and 75^th ^percentiles, of the antibody levels were summarized overall, and for the categories of the descriptive variables. Comparisons of the antibody levels across the different categorizations of the descriptive variables were achieved via analysis of variance (ANOVA), with rubella antibody levels being analyzed on the logarithmic scale to correct for data skewness. All analyses were adjusted for the following set of covariates potentially associated with the measured rubella-specific antibody titers: age at enrollment, race, gender, age at first rubella vaccination, age at second rubella vaccination, and cohort status.

While the focus of this effort was on the multigenic contributions of these SNPs to variation in rubella-specific antibody levels, a test of association was obtained for each genotyped SNP that met quality control criteria in our study subjects to examine the ordinal genetic association between each of the SNPs of interest while adjusting for the descriptive variables using linear regression. A quantile plot of the overall distribution of single-SNP p-values was extracted and summarized for all SNPs.

The primary interest was to identify the degree to which combinations of SNPs might provide novel insight into the potential control of rubella-specific antibody levels. Therefore, a series of multigenic analyses were carried out. We first assessed the degree to which individual SNPs provided additive information into the levels of rubella-specific antibody titers. We applied two approaches to identify these multi-allelic associations. The first was a single omnibus test of association where we identified the positively associated allele of each SNP and tallied the number of positively associated alleles of the candidate SNPs within each individual and assessed the association between this count variable and rubella antibody levels while controlling for the descriptive variables. The second approach again forced the inclusion of descriptive covariates, but relied on a stepwise model selection paradigm for identification of SNPs to be included. In this stepwise process, we added or removed a single ordinal SNP variable at a time until only SNPs that contributed to an association with rubella antibody levels remained in the regression model, using p-values for entrance or removal of 0.05. For each of these methods, we applied randomization tests to account for the fact that we identified the "positively associated allele" for each SNP within the same dataset in which we were performing the test of significance, and to account for the model selection approach we used to identify the model of candidate SNPs [39]. These randomization procedures rely on repeated random re-assignment of observed antibody levels to model the situation where there is no association between SNPs and phenotype. These repeated randomization data sets are then used to perform the analyses that were carried out on the original data. The resulting p-values from these randomized data sets are then used to obtain an empirical estimate of the null distribution of p-values. The final randomization p-value reflects how extreme the observed p-value is, relative to this null empirical distribution.

In order to search for potential combinations of SNPs that were jointly associated with antibody levels, we applied the approach advocated by Marchini et al [38], in which we enumerated all possible combinations of the genotypes observed for each pair of candidate SNPs and tested for differences in the log-transformed rubella antibody levels among these categories using linear regression methods while adjusting for the descriptive variables. After performing this test for each of the 263,175 pairs of candidate SNPs, we produced a quantile plot to summarize the difference between the observed p-value distribution and p-value distribution that would have been expected if there were no pairs of SNPs that were significantly associated with antibody levels.

Additionally, we used recursive partitioning techniques to search for potential higher-order interactions among the candidate SNPs that might influence the observed levels of rubella-specific antibody levels. For a quantitative phenotype, these methods recursively search for optimal combinations of splits of explanatory variables such that the differences in the average levels of the phenotype among the identified groups are maximized [47,48]. We utilized the recursive partitioning routines available in S-Plus (TIBCO Software Inc., Palo Alto, CA) to perform these assessments. All statistical tests were two-sided and, unless otherwise indicated, analyses were carried out using the SAS software system (SAS Institute, Inc., Cary, NC).

## Conflict of Interest Statement

Dr. Poland is the chair of a safety evaluation committee for novel non-rubella vaccines undergoing clinical studies by Merck Research Laboratories.

## Authors' contributions

VSP participated in conception and design of the study. He oversaw statistical analyses, prepared the initial draft of the manuscript and finalized the manuscript after the writing and editing that was done by the co-authors. RAV participated in the conception and design of the study. He participated in the statistical analyses and made critical revisions to the manuscript for important intellectual content focused on statistical analysis and interpretation of the results. MMO performed statistical analyses and critically revised the manuscript for important intellectual content focused on statistical analysis. IGO participated in the selection of candidate genes and SNPs. She critically revised the manuscript for important intellectual content, focusing primarily on the genetic and immunologic aspects. GAP participated in conception and design of the study. He oversaw study conduct as the holder of the grants that supported the research effort. He provided critical revisions to the manuscript for important intellectual content, focusing primarily on the broad implications of the research efforts. All authors have read and approved the final manuscript.

## Authors' Background

VSP Dr. Pankratz is an Associate Professor of Biostatistics with training in statistical genetics. His interests lie in the study of genetic associations with a variety of phenotypes. He has worked with the Mayo Vaccine Research Group since 1998, studying the genetics of immune responses following vaccination.

RAV Mr. Vierkant is an Assistant Professor of Biostatistics with extensive experience in the design and analysis of genetic association studies. He has worked with the Mayo Vaccine Research Group since 1998, studying the genetics of immune responses following vaccination.

MMO Ms. O'Byrne is a Statistical Programmer Analyst in the Division of Biomedical Statistics and Informatics with extensive experience in analyzing data, and in preparing code to run a broad variety of statistical analyses, both novel and standard.

IGO Dr. Ovsyannikova is an Associate Professor of Medicine with a broad background in immunology and vaccinology. She has extensive experience in the design and conduct of genetics studies that focus on the contribution of candidate genes to differences in a variety of measures of post-vaccination immune response.

GAP Dr. Poland is a Professor of Medicine, Molecular Pharmacology & Experimental Therapeutics, Infectious Diseases. He has led the Mayo Clinic Vaccine Research Group since 1988 and helped to quantify the degree of genetic contribution to variation in a number of post-vaccination immune response measures. The efforts of the group he leads have focused in recent years on the study of genetic associations with a broad range of immune response measures raised by a variety of vaccines.

## Supplementary Material

Additional file 1 Supplementary Table: Genes included in the analysis, along with the numbers of genotyped SNPs of various types. Click here for file
