# Supplementary material for: Associations between SNPs in candidate immune-relevant genes and rubella antibody levels: a multigenic assessment
Source: BMC Immunol. 2010 Oct 5;11:48. doi: 10.1186/1471-2172-11-48 (PMC2965704; doi:10.1186/1471-2172-11-48)
Supplement: Additional file 1 — Supplementary Table: Genes included in the analysis, along with the numbers of genotyped SNPs of various types. [file 1471-2172-11-48-S1.DOC]

| **Supplementary Table**. Genes included in the analysis, along with the numbers of genotyped SNPs of various types. | | | | | | | | |
| --- | --- | --- | --- | --- | --- | --- | --- | --- |
| Gene Symbol | 3'UTR | 5'UTR | coding | flanking 3'UTR | flanking 5'UTR | intron | UTR | Total SNPs |
| ACO1 | 0 | 0 | 0 | 1 | 0 | 0 | 0 | 1 |
| ADAR | 2 | 0 | 1 | 1 | 3 | 2 | 0 | 9 |
| CASP10 | 0 | 0 | 1 | 0 | 0 | 1 | 0 | 2 |
| CASP8 | 0 | 0 | 0 | 0 | 1 | 0 | 0 | 1 |
| CHRNB2 | 1 | 0 | 0 | 0 | 0 | 0 | 0 | 1 |
| CSF2 | 0 | 0 | 1 | 5 | 1 | 0 | 0 | 7 |
| CSF2RB | 0 | 0 | 1 | 7 | 7 | 3 | 0 | 18 |
| DDX58 | 1 | 1 | 3 | 0 | 1 | 11 | 0 | 17 |
| EIF2AK2 | 0 | 0 | 0 | 0 | 2 | 4 | 1 | 7 |
| FAM3B | 1 | 0 | 0 | 0 | 0 | 0 | 0 | 1 |
| FOXO4 | 0 | 0 | 0 | 0 | 0 | 1 | 0 | 1 |
| IFNA1 | 0 | 0 | 0 | 2 | 2 | 0 | 0 | 4 |
| IFNA2 | 1 | 0 | 0 | 3 | 1 | 0 | 0 | 5 |
| IFNA21 | 1 | 0 | 0 | 1 | 2 | 0 | 0 | 4 |
| IFNAR1 | 0 | 0 | 1 | 3 | 1 | 14 | 0 | 19 |
| IFNAR2 | 0 | 0 | 1 | 0 | 0 | 21 | 0 | 22 |
| IFNB1 | 0 | 0 | 0 | 4 | 6 | 0 | 0 | 10 |
| IFNG | 0 | 0 | 0 | 1 | 2 | 2 | 0 | 5 |
| IFNGR1 | 0 | 0 | 0 | 3 | 5 | 3 | 0 | 11 |
| IFNGR2 | 0 | 0 | 1 | 0 | 4 | 5 | 0 | 10 |
| IL10 | 2 | 0 | 0 | 4 | 7 | 3 | 0 | 16 |
| IL10RA | 1 | 0 | 2 | 1 | 0 | 5 | 0 | 9 |
| IL10RB | 1 | 0 | 1 | 9 | 0 | 6 | 0 | 17 |
| IL12A | 0 | 0 | 0 | 7 | 9 | 1 | 0 | 17 |
| IL12B | 2 | 0 | 0 | 5 | 5 | 6 | 0 | 18 |
| IL12RB1 | 0 | 0 | 3 | 2 | 0 | 15 | 0 | 20 |
| IL12RB2 | 0 | 1 | 0 | 0 | 2 | 19 | 0 | 22 |
| IL13 | 0 | 0 | 0 | 2 | 0 | 0 | 0 | 2 |
| IL18R1 | 1 | 0 | 0 | 3 | 3 | 9 | 0 | 16 |
| IL1A | 0 | 1 | 0 | 0 | 0 | 0 | 0 | 1 |
| IL1B | 0 | 0 | 0 | 0 | 1 | 0 | 0 | 1 |
| IL1RL1 | 0 | 0 | 0 | 2 | 0 | 0 | 0 | 2 |
| IL2 | 0 | 0 | 0 | 2 | 2 | 3 | 0 | 7 |
| IL2RA | 0 | 0 | 0 | 5 | 6 | 34 | 0 | 45 |
| IL2RB | 0 | 0 | 1 | 6 | 4 | 16 | 0 | 27 |
| IL2RG | 0 | 0 | 0 | 0 | 0 | 1 | 0 | 1 |
| IL3 | 0 | 0 | 0 | 4 | 0 | 0 | 0 | 4 |
| IL4 | 0 | 1 | 0 | 1 | 4 | 2 | 0 | 8 |
| IL4R | 1 | 0 | 5 | 5 | 3 | 14 | 0 | 28 |
| IL5 | 0 | 0 | 0 | 4 | 1 | 0 | 0 | 5 |
| IL6 | 0 | 0 | 0 | 4 | 8 | 1 | 0 | 13 |
| IL6R | 1 | 0 | 1 | 0 | 2 | 9 | 0 | 13 |
| IL6ST | 0 | 0 | 0 | 0 | 0 | 3 | 1 | 4 |
| IRF3 | 0 | 0 | 0 | 0 | 0 | 0 | 1 | 1 |
| IRF9 | 0 | 0 | 0 | 1 | 0 | 0 | 0 | 1 |
| ISG20 | 0 | 0 | 0 | 1 | 7 | 2 | 1 | 11 |
| ISG20L1 | 2 | 0 | 3 | 0 | 0 | 2 | 0 | 7 |
| KIAA1542 | 1 | 0 | 0 | 0 | 0 | 0 | 0 | 1 |
| LOC341333 | 0 | 0 | 0 | 1 | 0 | 0 | 0 | 1 |
| LOC442035 | 0 | 0 | 0 | 0 | 1 | 0 | 0 | 1 |
| LOC727988 | 0 | 0 | 0 | 3 | 0 | 0 | 0 | 3 |
| LST1 | 0 | 0 | 0 | 0 | 0 | 2 | 0 | 2 |
| LTA | 0 | 0 | 1 | 2 | 4 | 0 | 0 | 7 |
| MED12 | 0 | 0 | 0 | 0 | 2 | 0 | 0 | 2 |
| MUPCDH | 0 | 0 | 1 | 0 | 0 | 0 | 0 | 1 |
| MX1 | 1 | 0 | 2 | 4 | 1 | 12 | 0 | 20 |
| MX2 | 0 | 0 | 0 | 0 | 0 | 7 | 0 | 7 |
| OAS1 | 1 | 0 | 2 | 0 | 2 | 0 | 0 | 5 |
| OAS2 | 2 | 0 | 2 | 2 | 1 | 5 | 0 | 12 |
| OAS3 | 4 | 0 | 1 | 0 | 3 | 3 | 0 | 11 |
| RAD50 | 0 | 0 | 0 | 0 | 0 | 1 | 0 | 1 |
| RARA | 0 | 0 | 0 | 0 | 0 | 2 | 0 | 2 |
| RARB | 1 | 0 | 0 | 0 | 7 | 39 | 0 | 47 |
| RARG | 1 | 0 | 0 | 0 | 2 | 0 | 0 | 3 |
| REC8 | 0 | 1 | 0 | 0 | 1 | 1 | 0 | 3 |
| RGS16 | 0 | 0 | 0 | 1 | 0 | 0 | 0 | 1 |
| RNASEL | 0 | 1 | 2 | 3 | 1 | 0 | 0 | 7 |
| RNF31 | 0 | 0 | 0 | 0 | 0 | 1 | 0 | 1 |
| RPH3A | 0 | 0 | 0 | 3 | 0 | 0 | 0 | 3 |
| RXRA | 1 | 0 | 0 | 0 | 2 | 6 | 0 | 9 |
| SCNN1A | 0 | 0 | 0 | 1 | 0 | 2 | 0 | 3 |
| TLR3 | 0 | 0 | 1 | 1 | 3 | 3 | 1 | 9 |
| TLR4 | 1 | 0 | 0 | 4 | 4 | 3 | 0 | 12 |
| TMEM50B | 0 | 0 | 0 | 2 | 0 | 0 | 0 | 2 |
| TMPRSS2 | 2 | 0 | 0 | 0 | 0 | 0 | 0 | 2 |
| TNF | 0 | 0 | 0 | 0 | 1 | 0 | 0 | 1 |
| TNFRSF1A | 0 | 0 | 0 | 0 | 1 | 4 | 0 | 5 |
| TNFRSF1B | 4 | 0 | 1 | 0 | 1 | 20 | 0 | 26 |
| TOP2A | 1 | 0 | 0 | 2 | 0 | 1 | 0 | 4 |
| TOP2B | 0 | 0 | 0 | 0 | 0 | 3 | 0 | 3 |
| TRIM22 | 1 | 0 | 3 | 3 | 2 | 7 | 0 | 16 |
| TRIM5 | 0 | 0 | 2 | 0 | 1 | 1 | 1 | 5 |
| VDR | 0 | 0 | 0 | 1 | 3 | 9 | 1 | 14 |
| VISA | 1 | 0 | 1 | 0 | 1 | 0 | 0 | 3 |
| Totals | 40 | 6 | 45 | 132 | 146 | 350 | 7 | 726 |
